# BMC Medicine celebrates its 5^th ^anniversary

**DOI:** 10.1186/1741-7015-7-3

**Published:** 2009-01-15

**Authors:** Joanne M Appleford, Robin L Cassady-Cain, Jigisha Patel, Melissa L Norton

**Affiliations:** 1 BMC Medicine Editorial team, BioMed Central Ltd, Middlesex House, 34-42 Cleveland Street, London W1T 4LB, UK

## Abstract

In November 2008, *BMC Medicine *passed the landmark of its first 5 years of publishing. When we launched the journal with the aim of publishing high quality research of general interest and special importance, we had no idea what the future would bring. To mark the occasion of our 5^th ^anniversary, we consider the achievements of the last 5 years and discuss our plans for the future.

## Editorial

*BMC Medicine*, the flagship medical journal of the *BMC *series, recently celebrated its 5^th ^anniversary. Over the last five years, the journal has developed substantially and has even been accepted for citation tracking by ISI within its first three years of publishing. As a result we are eagerly anticipating the journal's first official Impact Factor, due in June 2009.

When *BMC Medicine *launched in November 2003, open access publishing was still a fairly novel concept. The last five years have seen open access evolve from what many considered to be a fringe movement to a widely accepted and sustainable business model for the publishing industry. This is particularly underlined by the recent acquisition of BioMed Central, the publisher of *BMC Medicine*, by Springer Science & Business Media, the world's second largest publisher of scientific, technical and medical journals.

*BMC Medicine *aims to publish articles of outstanding quality, broad interest and special importance. In 2003 we had no idea of what the future would bring-we launched *BMC Medicine *with the highest of expectations and the support of an internationally renowned Editorial Board, but would medical researchers accept such an open access journal?

Now, in celebration of our anniversary and in anticipation of our first official Impact Factor in June 2009, we reflect on the successes so far. As with all journals, the first measure of *BMC Medicine's *success is in the quality of its published articles. The systematic review of the incidence of schizophrenia, by John McGrath and colleagues [1], has been cited an amazing 92 times at the time of writing, and the second [2] and third [3] most cited articles have 52 and 40 citations respectively. *BMC Medicine *articles have been cited in such journals as *The Lancet, New England Journal of Medicine, JAMA *and *BMJ*, together with a whole range of other top tier general and subject-specific publications.

Another measure of quality is circulation – as *BMC Medicine *is an open access journal without subscription barriers or print editions, we use article accesses in place of circulation numbers. Most original research articles published in *BMC Medicine *become highly accessed [4] soon after publication, and the number of accesses over time can be staggering. For instance, the systematic review and meta-analysis of the incidence of contrast-induced nephropathy, by Sean Bagshaw and William Ghali [5], has been accessed almost 55,000 times from the *BMC Medicine *website alone. Moreover, an article by Chris McManus and colleagues [6] published this year has achieved over 10,000 article accesses in under 12 months. These articles are also freely available through a number of other sites, such as PubMed Central, so the actual numbers of accesses for each article are much higher.

We could not have achieved our strong readership and rapid development without the help and support of our international Editorial Board. Members of our Editorial Board have contributed their time as authors, referees, and advisors. We are indebted to them for all of these contributions, as well as the effort they put into raising the profile of the journal within their respective fields. Finally, we would not be where we are today without the significant contributions of our authors and reviewers: we look forward to your continued support as we move forward.

While the editorial staff are delighted with the journal's progress so far, we continue to strive to improve the quality of the content of *BMC Medicine*, our service to authors, and the position of the journal within the medical community. In line with these ambitions, we have substantial plans for development in the near-term and long-term future. We are expanding our portfolio of articles to include commissioned review and minireview articles, as well as commentaries on articles and subjects of interest to our readership. Other article types will follow in due course. In addition we are expanding the Editorial Board for *BMC Medicine*. 2009 will also see the Editorial Team attending conferences and meetings – we hope to meet with you soon!

We look forward to our next five years of publishing; in particular to receiving our first official Impact Factor, expanding the Editorial Board and publishing new content of interest to our readers. Of course, as ever, we anticipate publishing more research of excellent quality that changes medical research and clinical practice, together with thought-provoking and clinically relevant reviews to highlight recent developments in a given field.

If you are interested in contributing to *BMC Medicine *or have suggestions for how to improve the journal, please submit your article via the journal website  or contact the Editorial office with any queries editorial@biomedcentral.com. Our aim is to make *BMC Medicine *a leading general medical journal, and we look forward to working with you to accomplish this goal.

## Pre-publication history

The pre-publication history for this paper can be accessed here:


